# Quality of Life Assessment in Patients Using Benzodiazepines during the COVID-19 Pandemic in a Community Pharmacy Using EuroQol 5D-3L

**DOI:** 10.3390/pharmacy11010019

**Published:** 2023-01-18

**Authors:** Daida Alberto Armas, Juan Ramón Santana Ayala, Yanira Román Castillo, Arturo Hardisson de la Torre, Carmen Rubio Armendáriz

**Affiliations:** 1 Toxicology Department, Universidad de La Laguna, 38071 Canary Islands, Spain; 2 Community Pharmacy, 38010 Canary Islands, Spain; 3 Nuestra Señora de la Candelaria Hospital, 38010 Canary Islands, Spain

**Keywords:** benzodiazepines, pharmaceutical care, health quality

## Abstract

Users of benzodiazepines (BZDs) should have their quality of life monitored to minimize the risks associated with long-term treatments. The aim of this study is to use the EuroQol 5D-3L to analyze the quality of life of 127 patients under treatment with BZDs during the COVID-19 pandemic. The results show that lorazepam comprises 25.49% of all dispensing requests, and that the mean duration of BZDs treatments is four years (range: 0.3–25). When rating their general health status, BZDs users reported 59.29 points out of 100. Thirty-two percent of patients reported mobility problems; 16.5% reported having a lot of pain or discomfort despite being treated with BZDs, and 16.54% used a BZD together with an opioid analgesic. The EuroQol 5D-3L dimension “anxiety/depression” showed that, despite the use of BZDs, 48.2% of the patients reported being moderately anxious or depressed and 13.4% described themselves as very anxious or depressed. Nevertheless, 37.8% of BZDs users were identified as potential candidates to follow a BZD deprescription plan. In conclusion, BZDs users showed a low quality of life during the COVID-19 pandemic. Older patients and females have been identified as groups of patients that could benefit from integrating the use of the EuroQol 5D-3L instrument into the protocols of the pharmaceutical care follow up.

## 1. Introduction

Among the different therapeutic groups, psychotropic drugs stand out as some of the most dispensed drugs in community pharmacies. In 2014, a report by the Spanish Agency of Medicines and Health Products (AEMPS) on the use of anxiolytic and hypnotic drugs in Spain 2000–2012 reflected an increase of 46.8% and 81.8% in the anxiolytic and hypnotic and sedative groups, respectively [1]. According to recently published prescription consumption data for 2021 in Spain [1], prescriptions of anxiolytics and antidepressants have increased in the last year from 90,603 to 93,046 DHD (Defined Daily Doses per 1000 inhabitants per day), with a more striking increase with respect to 2019 (86,935 DHD). Finally, the Survey on Alcohol and Drugs in Spain [2] not only shows an increasing consumption of hypnosedatives, but reports a higher prevalence in women and in the 35–64 age group.

BZDs have been identified as one of the pharmacological groups with the highest prescription and dispensing rates [3,4,5,6,7,8]. The latest National Health Survey in Spain indicates that 1 in 10 Spaniards take BZDs [9].

BZDs are considered safe for short-term use; however, chronic use and higher doses than those recommended in Clinical Practice Guidelines (CPGs) and data sheets have been associated with several risks and adverse effects such as cognitive impairment, tolerance, dependence, falls related to hip fractures and traffic accidents, among others [10,11,12]. Given the high consumption figures and the risks associated with the use of BZDs, some strategies to reduce their prescription and consumption have already been developed and suggested in both primary care and community pharmacy [13,14,15,16].

Because quality of life is frequently affected in patients consuming BZDs, it is important to have tools and instruments to assess and monitor patients’ quality of life while they are receiving pharmaceutical care. Health-related quality of life (HRQoL) is a measure of an individual’s or a group’s perceived physical and mental health over time that increases, especially in chronic diseases, information about the opinions, preferences, and discomfort of patients. A negative association was identified between indicators of anxiety and HRQoL, suggesting that these variables are risk factors for quality of life [17]. The intervention of community pharmacists shows the potential to minimize negative impacts on the patient’s quality of life during the use of BZDs and, therefore, the measurement of quality of life has become increasingly relevant during the follow-up of these and other medicine users [18,19].

Among the questionnaires available to estimate quality of life, the EuroQol 5D-3L (Table 1) is the most widely used multiattribute utility instrument for measuring health-related quality (Little et al., 2014; EQ-5D-3L User Guide, 2018). The 3-level version of EQ-5D (EQ-5D-3L) was introduced in 1990 by the EuroQol Group and consists of two pages: the EQ-5D descriptive system and the EQ visual analogue scale (EQ VAS). The EQ-5D-3L descriptive system is comprised of the following five dimensions: mobility, self-care, usual activities, pain/discomfort, and anxiety/depression. Each dimension has three levels: no problems, some problems and extreme problems. The patient is asked to indicate his/her health state by ticking the box next to the most appropriate statement in each of the five dimensions. The EQ VAS records the patient’s self-rated health on a vertical visual analogue scale where the endpoints are labelled ‘Best imaginable health state’ and ‘Worst imaginable health state’. The VAS can be used as a quantitative measure of health outcome that reflects the patient’s own judgement from 0 (worst possible health status) to 100 (best possible health status) [20,21].

Considering this background, the aim of this study is to describe and analyze the quality of life of patients requesting the dispensation of a BZD at a community pharmacy over a period of six months during the COVID-19 pandemic, and to correlate this quality of life with the gender and age of the patients while receiving the pharmaceutical care dispensing service at the community pharmacy. Additionally, the profile of patients whose quality of life is affected by these drugs is characterized and pharmaceutical intervention is guided.

## 2. Materials and Methods

A prospective cross-sectional descriptive observational study (AEMPS code: DAI-LOR-2020-01), without a control group, was carried out for six months (August 2020–February 2021) in a community pharmacy in Tenerife (Canary Islands, Spain). One hundred and twenty-seven patients of both genders were included in the study considering the following criteria: 

Inclusion criteria: Patients starting or continuing treatment with BZD as a monodrug (lorazepam, lormetazepam, alprazolam, diazepam, bromazepam, clorazepate potassium, clonazepam, ketazolam, clobazam and flurazepam); patients aged between eighteen and ninety years of age; patients who agreed to voluntarily participate in the study and who signed the informed consent form; patients whose communication and/or decision-making abilities were not impaired; caregivers who go to the pharmacy to pick up a BZD prescribed for the patient they care for (a caregiver is defined as a person who is responsible for the acquisition and administration of medication for a dependent patient, whether or not they are a relative). 

Exclusion criteria: patients who, although fulfilling the inclusion criteria, did not agree to participate in the study; patients who did not agree to sign the informed consent form; patients prescribed with combinations of BZDs or other active ingredients; patients not evaluable for a variety of reasons, at the discretion of the researcher, including incomplete records, suspicion of transcription errors in the database, unverified suspicion of exclusion criteria, among others; patients with communication, psychological or linguistic difficulties or without decision-making capacity; patients who decided to leave the study voluntarily; pregnant or breastfeeding women; patients referred from other professional pharmaceutical care services, as they may skew the results, given that patients would have received personalized information about their medication in each of these services, so their knowledge may be greater than that of patients who have not received this and the data may contain a bias.

Each patient agreed to participate voluntarily and signed an informed consent form. Data collection was performed by means of a structured clinical interview in the personalized pharmaceutical care area of the pharmacy. A questionnaire including the Eurool 5D-3L quality of life instrument and other variables such as sociodemographic variables, type of BZD, and duration of BZD treatment was used. 

The sample size corresponds to an acceptable level with a confidence interval of 95% and an estimated accuracy of 5%. The statistical analysis consisted of, firstly, a description of the participants, including 95% confidence intervals (CI), and secondly, a correlation study with comparative tests of means (Pearson’s chi-square and ANOVA), considering significance *p* < 0.05, to study the associations between the dimensions of the EuroQol 5D-3L quality of life instrument and the rest of the variables mentioned above. Data analysis was performed using SPSS 25.0™ software from IBM Co.^®^ (Armonk, NY, USA) on a Windows NT 365 Professional™ operating system from Microsoft Co.^®^ (Redmond, WA, USA).

## 3. Results and Discussion

The gender distribution of the sample was 66.14% women and 33.86% men (two out of three BZDs users are women). These results are in line with previous studies conducted in Spain such as those of García et al. [22] who studied 78% women, García-Delgado et al. [15] with 71%, Toral-López et al. [8] with 66%, Matud et al. [23] with 52.6% and García et al. [24] with 62% women. Furthermore, studies in which only patients over 65 years of age were assessed also reported a higher percentage of women (74%) among BZDs users [12]. The mean age in the studied population (61 years, range 20–89) is similar to that observed in different national studies [8,24,25,26]. This mean age is associated with a special vulnerability to psychiatric disorders susceptible to treatment with BZD [27].

The most frequent BZD among the ten molecules studied is lorazepam with 25.49% of all dispensing requests, followed by diazepam with 14.38%, lormetazepam with 14.38%, alprazolam with 13.73% and clorazepate with 12.42%. Bromazepam and clonazepam have lower percentages of dispensing requests with 8.50% and 5.88%, respectively. Finally, with the smallest percentages, we find ketazolam with 1.96%, flurazepam with 1.96% and clobazam with 1.31%, which reflects their low use as a therapeutic resource in our community.

The mean duration of the BZDs treatments in the patients studied was four years (range: 0.3–25). Such a long duration of treatment is identified as a risk for the patient, because according to the Clinical Practice Guidelines (CPGs) and technical data sheets of the different BZDs, the duration should not be extended beyond twelve weeks, including the withdrawal period. The chronicization of BZDs treatments has been previously observed by other authors. Baza et al. [25] also observed 34% of patients with more than four years of BZD treatment, and Velert et al. [12] and Sake et al. [28] reported that 67% of BZDs users had been on treatment for more than one year. These results suggest that any pharmaceutical intervention during dispensing of BZDs should consider the review and reassessment of the BZD treatment by the prescribing physician, with deprescription being the best option.

The visual analogue scale of the EuroQol 5D-3L test allowed the BZDs patient to describe his or her current health status with a score between 0 and 100. The results obtained show a mean score (range) and a standard deviation of 59.29 (10–90) ± 21.20. This result is lower than expected because BZDs, due to their high effectiveness in counteracting symptoms, should provide a rapid response to the health problem for which they are prescribed. In the Spanish National Health Survey (ENS) 2011–2012, the EuroQol test reaches figures of 77.53 points on average at a national level and 76.02 points in the Canary Islands [29], with both values being higher than those obtained in the present study. The differences of almost twenty points between the results of the present study and those of the ENS are explainable not only on the basis that the study here was carried out on a sick population of subjects consuming BZDs, but also because the present study was carried out during the COVID-19 pandemic when, in general, the population was exposed to such uncertainty that their perception of their state of health was negatively impacted. In the Spanish study conSIGUE [30], mean figures of 63.8 points were obtained, which can be considered similar to that of the present study. Martínez-Martínez et al. [31], with the adherenceMED project, obtained a mean value of 68.55 points, somewhat higher than that of the study here.

**Figure 1:**
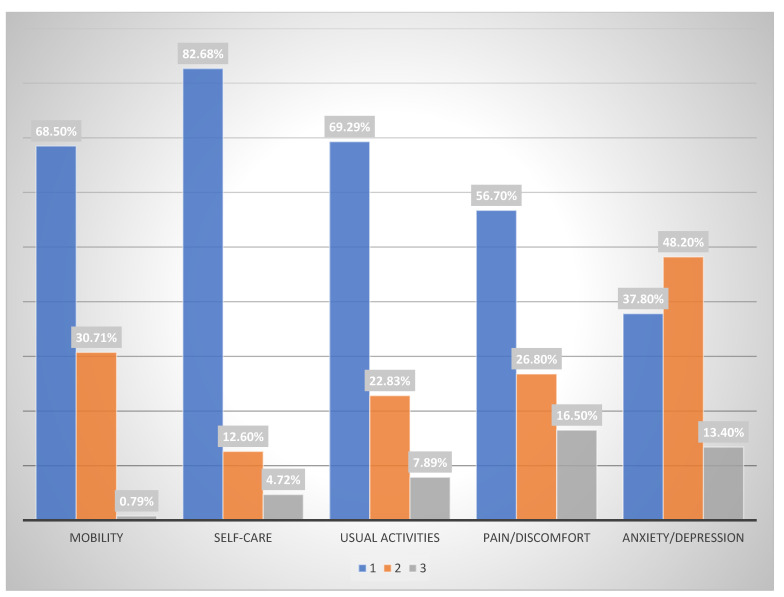
Distribution of responses to the EuroQol 5D-3L instrument in BZDs users at the dispensing service in the community pharmacy. For each item, the score 1 to 3 indicates the ascending level of severity.

The EuroQol 5D-3L test provides information on the patient’s health status. The results obtained for the different dimensions included in the EQ-5D-3L descriptive system section of the EuroQol 5D-3L test are shown in Figure 1.

Regarding mobility and associated problems, 68.50% of BZDs users indicated that they had no problems walking, 30.71% reported some problems with walking, and 0.79% reported the need to be in bed (Table 2). The high percentage of patients responding that they had some walking difficulties is in line with the results reported by Hernández et al. [32] who detected a similar score of 37.1%. However, García et al. [22], when studying the BZD-using population and measuring their mobility, observed that 26.5% of men and 22.9% of women reported having some problems with walking. Having obtained a total of almost 32% of patients with mobility problems, the authors proceeded to investigate the correlation with age (Table 2). The statistical association showed that the mean age of the patients with walking problems was sixty-nine years of age, which is twelve years higher than that of patients with no walking problems (*p* = 0.003), which is statistically significant. This result is consistent and expected, because patients in this age bracket usually present greater mobility difficulties.

In the self-care quality-of-life parameter, the EuroQol instrument showed that 82.68% of BZDs users reported having no problems with self-care, 12.60% had some problems with self-care, and 4.72% were unable to wash or dress themselves. García et al. [22] obtained similar figures with 91.7% having no problems with self-care (82.68% in the present study) and 8.1% having some problems (12.60% in the present study). With respect to usual activities, 69.29% of BZD users had no problems with carrying out daily activities, 22.83% had some problems, and 7.87% were unable to carry out their daily activities. In relation to the pain and discomfort dimension, 56.7% of BZD users reported having neither, 26.8% reported moderate pain or discomfort, and finally, 16.5% reported having a lot of pain or discomfort despite being treated with BZD. The study here therefore shows that almost half of the BZD-using population had some degree of pain and discomfort.

Comparing these results with those reported by García et al. [22], it can be concluded that the data are quite similar. Hernández et al. [32] obtained a figure of 57.1% for pain/discomfort for polymedicated patients which is also similar to that obtained in the present study. When evaluating gender, it was found that those patients with no pain were 57.8% male and 42.9% female, those with moderate pain and discomfort were 57.8% male and 46.8% female, and those with a lot of pain or discomfort were 4.8% male and 10.3% female. 

According to data from the European Health Survey for Spain (2020), among the most frequent chronic problems in people over fifteen years of age, chronic lumbar back pain was the third most common problem. In the case of people over sixty-five years of age, data from the latest Spanish National Health Survey [9] show that 15% of people in this age group suffer severe or extreme pain (9% in men and 21% in women). In the present study, for the same group of patients as the national survey, given that the mean average age in the study population is sixty-one years of age, 16.5% of BZD users responded in the EuroQol as suffering a moderate to severe degree of pain, which is a result very much in line with that reported for the Spanish population (15%).

With the aim of characterizing BZD users, the authors sought to correlate this result with BZDs concomitant treatments. The results showed that 16.54% of the patients used a BZD together with an opioid analgesic. This combined use of these two drug types exposes the patient to the potential risk of suffering an unsafe interaction. In these cases, the pharmacist’s intervention should be to verify this potential drug interaction, investigate whether the patient shows signs of negative health outcomes, and refer the patient to the doctor if necessary.

The last dimension of the EuroQol 5D-3L instrument concerns the assessment of anxiety and depression. Although the sample studied should have had their anxiety and depression minimized, the results show a high prevalence of signs and symptoms of anxiety and depression among the patients studied. A total of 13.4% of patients reported being very anxious or depressed, 48.2% were concerned about being moderately anxious or depressed, and 37.8% reported being neither anxious nor depressed. Hernández et al. [32] obtained slightly lower but equally remarkable data (41.1%). García et al. [22] reported a result of 42.5% of BZD users with no depression or anxiety, 45.8% in a moderate state and 10.7% in the most severe degree. As both studies show, the values are similar, especially in the higher degrees of anxiety or depression.

**Figure 2:**
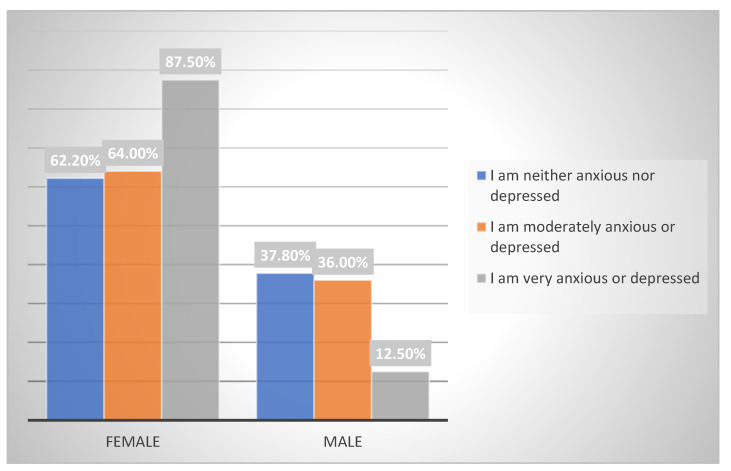
Statistical parameters of the gender–anxiety/depression association in BZD users. % according to gender (female: 66.7% vs male: 33.3%).

There is a significant proportion of BZDs patients in whom symptoms of anxiety and depression persist. Statistical analysis of the relative frequency of the group of patients showing some degree of anxiety and depression gives a result of 53.39–70.61%, with a 95% CI. These numbers are higher than expected and may be explained by the tolerance caused by BZDs. This was also observed in the study by García et al. [22] where, in the moderately anxious or depressed and very anxious or depressed categories, women scored 47.8% and 12%, respectively (Figure 2). Although the methodology of the study by García et al. [22] is different from that of the present study, both studies confirm the higher prevalence of anxiety and depression in the female gender, although statistical significance was not attained in the present study (*p* = 0.158).

When exploring the possible relationship between anxiety/depression and age, it was observed that the distribution between the age of the patients and their state of anxiety or depression followed the same pattern in the three categories of the EuroQol instrument (Table 3, with the mean age for the three categories being sixty years of age. No statistical significance was found after an ANOVA test of the age difference for the age–anxiety and depression condition (*p* = 0.963)).

The authors believe that in many cases, the health problem which the BZD was initially prescribed for may not be receiving a proper follow-up, and this deficiency may be generating potentially inappropriate prescriptions (PIP). In these cases, if detected at the dispensing service, the pharmacist’s intervention should consist of referring the patient to the prescribing doctor proposing a readjustment or modification of the BZD treatment or information about other pharmacological alternatives. In BZDs patients with some degree of anxiety and depression, collaborative teamwork should be promoted by the health services between prescribing doctors and dispensing pharmacists. The pharmacist’s intervention is of great importance, because the dispensing service allows the assessment of BZD health outcomes while observing the patient’s emotional state and concern about the lack of efficacy of the BZD.

Nevertheless, among the different healthcare providers’ interventions in long term BZDs patients, referral to cognitive behavioral therapy (CBT) stands out, along with personalized health education in all healthcare settings, and advice about life habits such as relaxation strategies or sleep hygiene, among others.

In the case of the 37.8% of BZDs patients who reported not being anxious or depressed, the authors conclude that the combined use of the BZD together with other associated drugs achieve the therapeutic objective of controlling these health problems. Therefore, this 37.8% of patients is identified by the community pharmacy as an appropriate population to follow a process of BZD deprescription as recommended, to minimize the risks associated with long-term BZD treatments.

## 4. Conclusions

According to the EuroQol 5D-3L instrument, the mean score of perceived health status is low in BZDs users. Because female BZDs users are the group of patients with the lowest scores on the EuroQol 5D-3L dimensions, they can be identified as being vulnerable patients and a potential target for pharmaceutical care services. The proportion of patients over sixty-five years of age on BZDs treatment who show difficulty in mobility, pain/discomfort and depression/anxiety according to the EuroQol 5D-3L dimensions should be addressed by different healthcare providers including the community pharmacists. There is a need and an opportunity for tailored pharmaceutical care that optimizes the safe and effective use of BZD and improves awareness of BZD among users. During dispensation of BZDs, the use of the EuroQol 5D-3L instrument allows the detection of those users who continue to perceive anxiety and/or depression and need to have their BZDs treatments reassessed. The pharmacist’s knowledge of this EuroQol 5D-3L health standard facilitates pharmaceutical intervention and decision-making at the community pharmacy care practice. Finally, these results reflect the need to enhance the physician–patient–pharmacist link.

Limitations: One of the limitations of the study may be related to the final sample size. Nevertheless, as detailed in the statistical procedure, we have managed to maintain statistical significance. Furthermore, all cross-sectional studies have some limitations, especially when compared with cohort or case-control studies that compare results over a period of time. However, it is worth noting the strength of cross-sectional studies in terms of time and minimal cost.

## Figures and Tables

**Table 1:** EuroQol 5D-3L for quality of life [EuroQol Research Foundation, 2018].

Test EuroQol 5D-3L
**1. MOBILITY** -I have no problems walking -I have some problems walking -I have to stay in bed
**2. SELF-CARE** -I have no problems with personal care -I have some problems with washing or dressing myself -I am unable to wash or dress myself
**3. USUAL ACTIVITIES** -I have no problems in carrying out my daily activities. -I have some problems in carrying out my daily activities. -I am unable to carry out my daily activities.
**4. PAIN/DISCOMFORT** -I have no pain or discomfort -I have moderate pain or discomfort -I have a lot of pain or discomfort
**5. ANXIETY/DEPRESSION** -I am neither anxious nor depressed -I am moderately anxious or depressed -I am very anxious or depressed

**Table 2:** Correlation between mobility and age in patients using BZDs.

Group Statistics								
	Mobility	N	Mean	Standard Deviation	Standard Error of Mean	95% Confidence Interval for Difference		Significance. (Bilateral)
Age (years)	I have no problems walking	82	57.20	15.272	1.686	Lower	Higher	
	I have some problems walking	29	68.45	9.720	1.805	−16.171	−6.335	0.003

**Table 3:** Statistical parameters of the age–anxiety/depression association in BZD users.

Age (Years)					
I am neither anxious nor depressed		N	Valid	45	
		Mean		60.18	
		Standard deviation		1.714	
I am moderately anxious or depressed		N	Valid	50	
		Mean		59.82	
		Standard deviation		14.872	
I am very anxious or depressed		N	Valid	16	
		Mean		61.00	
		Standard deviation		12.987	
One-factor ANOVA					
Age (years)					
	Sum of squares	gl	Mean square root	F	Significance
Inter-groups	17.015	2	8.508	0.038	0.963
Intra-groups	24,231.958	108	224.370		
Total	24,248.973	110			

## Data Availability

Not applicable.
